# Antinociceptive, Anti-Inflammatory, and Antipyretic Activity of Mangrove Plants: A Mini Review

**DOI:** 10.1155/2012/576086

**Published:** 2012-05-17

**Authors:** J. A. Shilpi, M. E. Islam, M. Billah, K. M. D. Islam, F. Sabrin, S. J. Uddin, L. Nahar, S. D. Sarker

**Affiliations:** ^1^Pharmacy Discipline, Khulna University, Khulna 9208, Bangladesh; ^2^Biotechnology and Genetics Discipline, Khulna University, Khulna 9208, Bangladesh; ^3^Department of Biotechnology and Genetic Engineering, Mawlana Bhashani Science and Technology University, Santosh, Tangail 1902, Bangladesh; ^4^School of Pharmacy, Griffith University, QLD 4222, Australia; ^5^Leicester School of Pharmacy, De Montfort University, The Gateway, Leicester LE1 9BH, UK; ^6^Department of Pharmacy, School of Applied Sciences, University of Wolverhampton, MA Building, Wulfruna Street, Wolverhampton WV1 1LY, UK

## Abstract

Mangrove plants are specialised plants that grow in the tidal coasts of tropic and subtropic regions of the world. Their unique ecology and traditional medicinal uses of mangrove plants have attracted the attention of researchers over the years, and as a result, reports on biological activity of mangrove plants have increased significantly in recent years. This review has been set out to compile and appraise the results on antinociceptive, anti-inflammatory, and antipyretic activity of mangrove plants. While the Web of Knowledge, Google Scholar, and PubMed were the starting points to gather information, other pieces of relevant published literature were also adequately explored for this purpose. A total of 29 reports on 17 plant species have been found to report such activities. While 19 reports were on the biological activity of the crude extracts, 10 reports identified the active compound(s) of various chemical classes of natural products including terpenes, steroids, and flavonoids. This review finds that antinociceptive, anti-inflammatory, and antipyretic activity appears to be widespread in mangrove plants.

## 1. Introduction

Mangrove forests are a special type of vegetation found in the coastal regions of the tropical and subtropical parts of the world. Global area that comprises mangrove forest is about 181000 square km. Majority of the mangrove forests is confined to the South East Asia and Australia, which accounts for 43% of the worldwide mangrove area (Table 1) [1, 2]. About 70 plant species of 27 genera have been reported from mangrove forests [2]. However, it should be noted that mangrove forests generally support the growth of non-mangrove plant species as well. For example, 334 plant species of 245 genera have been reported so far from the Sundarbans [3]. Flora of mangrove forests is unique from others in that their habitat extends along the border where the fresh and sea water merge. Therefore, unlike common terrestrial plants, they can withstand high salt concentration, can remain submerged in water, and maintain an efficient nutrient retention mechanism [1].

Mangrove forests are still quite unfamiliar to a vast population due to their limited distribution. However, the people inhabiting areas near mangrove forests heavily depend on these forests to meet their needs including their healthcare. During the early stage of human civilization, mangrove forests drew very little or no attention. This is to some extent because of the difficulty to access these areas. As the population continued to grow, people had to find new and unexplored sources including mangrove forests. In some parts of the world, mangrove forests are over utilised. As a result, human establishment grew in close proximity of these forests. For example, the density of population near the Sundarbans is as high as >500 per sq km [2]. Most of these people are directly or indirectly rely on the Sundarbans for their livelihood. In addition, natural disasters are putting these forests under the threat of extinction. For example, the mangrove forest in Tamil Nadu State of India was declared Reserve forests in 1880, but its protection ultimately failed [2].

Like other terrestrial plants, many mangrove plants have ethnopharmacological relevance and have also been exploited by the local people in the search for remedies for various ailments. However, only a few of the mangrove plants have so far been included in any books listing medicinal plants. This may be due to the difficulty in collecting and identifying these plant species and lack of adequate information available about their uses. As a part of our INSPIRE Project, funded by the British Council, a recent visit to the Sundarbans and subsequent interviews with people living nearby villages have revealed that the local people use a number of plants from the Sundarbans to treat various medical conditions.

With the introduction of rapid and reliable screening methods, researchers around the world have picked plant species of various origins including mangrove plants in the search for new medicine. This review aims to compile and appraise reports on the antinociceptive, anti-inflammatory, and antipyretic activity of mangrove plants.

## 2. Methodology

Web of knowledge, Google Scholar, and PubMed were used to search for the published reports since 1950. Other relevant publications, for example, books and journal articles, were also consulted. A total of 57 mangrove species were searched for the activity. The results are presented in three different tables; Table 2 gives a general outline of works that have been carried out so far on various mangrove plants for antinociceptive, anti-inflammatory, and antipyretic activity. It also describes the plant species, family, plant part used for the investigation, reported activity, and the screening method.  Table 3 deals with those reports reporting the identification of active compound(s).

## 3. Antinociceptive, Anti-Inflammatory, and Antipyretic Activity

From the search, 29 hits were found with different mangrove species reporting one or more of these activities: antinociceptive, anti-inlfammatory, and antipyretic activity (Tables 2 and 3) [4–32]. Some of the reports coincide for a given species, and, therefore, a total of 17 plants were reported to have such activity. However, only one plant, *Pongamia pinnata* was studied for antipyretic activity. In nine cases, further phytochemical studies were carried out to find out the active constituent(s). One of the studies justified that the activity might be due to betulinic acid since betulinic acid is known for its anti-inflammatory activity and was present in the extract [8]. According to chemical classification, the active compounds, isolated from the mangrove plants, can be classified into diterpenes [11, 15], flavonoids [24], isoflavonoids [25, 29], monoterpenes [30], phenolics [30], steroids [32], triterpenes [29], xanthones [14], and a compound with unidentified structure [13] (Table 3).

**Figure 1:**
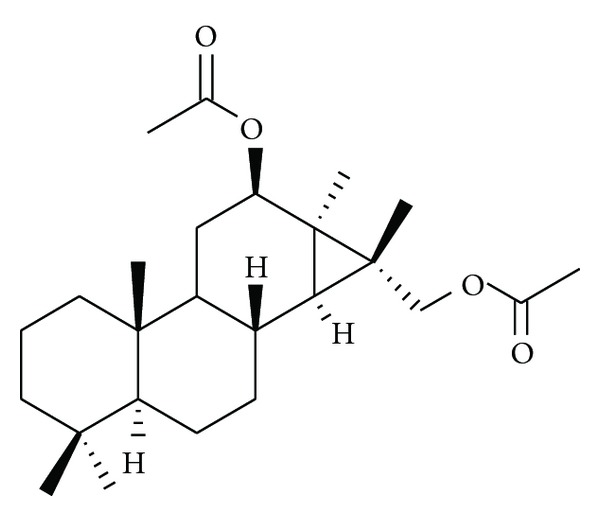
Mimosol D, an anti-inflammatory diterpene from the roots of *Caesalpinia mimosoides. *

**Figure 2:**
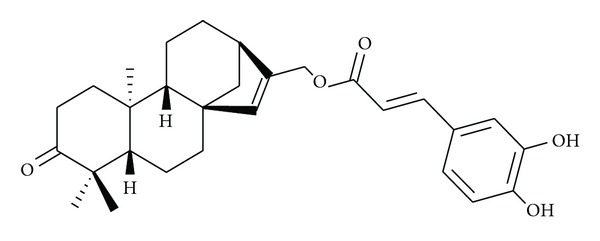
Agallochaol O, an anti-inflammatory diterpene from the stems and twigs of *Excoecaria agallocha. *

**Figure 3:**
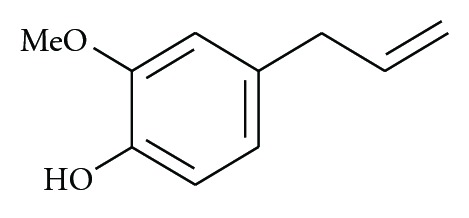
Eugenol, an analgesic and anti-inflammatory compound, from *Ipomoea-pes-caprae. *

The diterpenoids reported by Yodsaoue et al. [11] from the root extract of *Caesalpinia mimosoides* showed anti-inflammatory activity in micromolar range. The most potent activity was observed with mimosol D (Figure 1), which showed an IC_50_ for the inhibition of nitric oxide production at 3 *μ*M and TNF-*α* production at 6.5 *μ*M. Among the diterpenoids from the stems and twigs of the Chinese mangrove plant, *Excoecaria agallocha*, agallochaol O (Figure 2) at 100 *μ*M showed 52.6% inhibition of interleukin-6 (IL-6) and other proinflammatory cytokines induced by lipopolysaccharide (LPS) [15]. Bio-assay guided phytochemical investigation of *Ipomoea-pes-caprae* resulted in the isolation of eugenol (Figure 3), a well-known analgesic, anti-inflammatory natural product [31, 33]. Some studies resulted in the isolation of steroids and triterpenes as the active compounds (Table 3) [32].

Plants often produce secondary metabolites under stressful conditions. Therefore, it is not surprising that mangrove plants, facing various ecological and environmental stresses, biosynthesise a wide range secondary metabolites of potential medicinal importance. The present literature survey has revealed that mangrove plants contain a wide range of compounds showing antinociceptive, anti-inflammatory and or antipyretic activity (Tables 2 and 3).

Pain itself is not any disease. It is manifested in certain disease or pathological conditions. Use of natural products in the management of pain goes back to thousands of years. Use of poppy by various civilizations or the use of willow bark to cure fever led to the isolation of morphine and salicylic acid, respectively [34]. These two drugs are still used extensively in modern medical practice. Present trend of the researchers to focus on mangrove plants has opened up an arena to find bioactive compounds from a source that has long been ignored or less explored. It is expected that research on mangrove plants will continue to rise in the coming days.

## 4. Possible Mechanism of Actions

It must be stressed that there are no or a few reports available on the possible mechanisms of action of the extracts or isolated compounds from the mangrove plants. However, exploring the methods applied in the published reports on evaluation of antinociceptive, anti-inflammatory, and/or antipyretic activity of mangrove plants [4–34], the following assumptions can be made about the possible mechanisms of actions. The sensation of pain can be initiated either peripherally or through the central nervous system. Peripherally mediated pain can be inhibited by NSAIDs which blocks the anti-inflammatory pathways responsible for pain. On the other hand, opioid analgesics are useful for the management of centrally acting pain in which opioid analgesics act by inhibition of opioid receptors. Acetic-acid-induced and formalin-induced paw licking represents peripherally acting pain sensation. Intraperitoneal administration of acetic acid or formalin mediates pain response through the release of inflammatory mediators, mainly prostacycline (PGI_2_) [35, 36]. The hot plate test, the tail flick test, and the Randall-Selitto nociceptive test represent nociception through central mechanism [35, 37]. The rat paw oedema is an anti-inflammatory model that can be induced by carrageenan, formalin, kaolin, cotton pellet granuloma and granuloma, pouch. Inflammation of the rat paw can also be stimulated by administration of inflammatory mediators like histamine, or eicosanoids like 5-hydroxytryptamine and prostaglandin E-2 [22, 25]. Other anti-inflammatory models that have been used in the assessment include nitric oxide, TNF-*α*, and IL-6 induction by the administration of lipopolysaccharides in cell culture [14].

A wide range of methods were adopted by different research groups for the study of antinociceptive activity of mangrove plants. All these methods can be summed up to two major mechanisms, that is, centrally acting and peripherally mediated pain sensation. Different mangrove plants were able to inhibit pain sensation of both types. Therefore, it is possible to find opioid analgesics as well as analgesics in mangrove plants that act by inhibition of inflammatory pathways responsible for pain. Only in few cases, plants were investigated by methods that represent both of the mechanisms. Interestingly, articles that report the isolation of active compounds used methods representing peripherally acting pain sensation.

## 5. Conclusions

This review has revealed that antinociceptive, anti-inflammatory, and antipyretic activity appears to be widespread among mangrove plants, and thorough and systematic phytochemical and pharmacological studies are much needed to discover new antinociceptive, anti-inflammatory, and antipyretic medicinal entities from mangrove plants.

## Figures and Tables

**Table 1:** Distribution of major mangrove forests around the world [2].

Region	Country
South and South East Asia	The Sundarbans, Bangladesh and India; Pichavaram, India; Balochistan, Pakistan; Estuarine mangroves, Thailand; Srilanka; The Philippines; East China, Taiwan; Japan; Malaysia; Borneo, Java and Eastern Indonesia
Middle East	Arabian Peninsula; Red Sea; Gulf including Bahrain, Qatar, UAE and Oman
Australasia	Western and Eastern Australia; South Pacific Islands; Papua New Guinea; Solomons Island
North and South America and the Caribbean	Florida and Bahamas, USA; Mexico; Puerto Rico; Eastern Venezuela; Trinidad; Guiana, Brazil
Africa	North West of Africa stretching from Mauritania to Sierra Leone; West of Africa from Liberia to Nigeria; South West Africa from Nigeria to Angola; East of Africa from Somalia to Tanzania; Mozambique; Madagascar and South Africa

**Table 2:** Antinociceptive, anti-inflammatory, and antipyretic activity of mangrove plant species.

No	Plant name	Family	Plant part tested	Observed activity	Test method	Refs
1	*Acanthus hirsutus* Boiss.	Acanthaceae	Aqueous extract	Antinociceptive	Acetic-acid-induced in mice	[4]
2	*Acanthus ilicifolius* Linn.		MeOH fraction of leaf extract	Anti-inflammatory	Carrageenan-induced rat paw oedema, COX (1 and 2) and 5-LOX activity	[5]

3	*Aegiceras corniculatum* (Linn.) Blanco.	Myrsinaceae	*n*-Hexane, EtOAc and MeOH extracts of stem	Antinociceptive, Anti-inflammatory	Acetic-acid-induced, formalin-induced paw licking and hot plate test in mice	[6]
4	*Aegiceras corniculatum* (Linn.) Blanco.		MeOH extract of stem	Anti-inflammatory	Rat paw oedema and peritonitis models were employed for *in vivo* studies. For *in vitro* studies, human platelets and rat neutrophils were stimulated with Ca(2+)-ionophore A23187 leading to the production of various proinflammatory metabolites, that is, 12-HTT, 12-HETE and LTB(4), and 5-HETE	[7]

5	*Avicennia officinalis *Linn.	Avicenniaceae	MeOH extract of leaves	Anti-inflammatory	Freunds adjuvant-induced arthritis, carrageenan-, and formalin-induced rat paw oedema	[8]

6	*Barringtonia racemosa* Linn.	Lecythidaceae	98%* n*-Hexane, 98% CHCl_3_ and 95% EtOH extracts of leaf	Anti-inflammatory	Inhibition of nitric oxide formation in RAW 264.7 cells by Griess assay Amount of lipid peroxidation by ferric thiocyanate method	[9]
7	*Barringtonia racemosa* Linn.		Aqueous bark extract	Antinociceptive	Tail flick, hot plate, and formalin tests in rat	[10]

8	*Caesalpinia mimosoides* Lamk.	Leguminosae	CH_2_Cl_2_ and acetone extracts, pure compounds	Anti-inflammatory	Inhibition of lipopolysaccharide (LPS) induced nitric oxide (NO) production in RAW 264.7 cell lines	[11]

9	*Ceriops decandra *(Griff.) W. Theob.	Rhizophoraceae	EtOH extract of leaf and pneumatophore	Antinociceptive	Acetic-acid-induced in mice	[12]

10	*Calophyllum inophyllum* Linn.	Clusiaceae	EtOH extract of nut kernel	Anti-inflammatory	Carrageenan- and formalin-induced rat paw oedemas, cotton pellet implantation	[13]
11	*Calophyllum inophyllum* Linn.		(Pure compounds tested)	Anti-inflammatory	Carrageenan-induced hind paw oedema, cotton pellet granuloma and granuloma pouch techniques, in normal and adrenalectomized rats	[14]

12	*Excoecaria agallocha* Linn.	Euphorbiaceae	(Pure compounds tested)	Anti-inflammatory	Suppression of the expression of NF-*κ*B and AP-1 targeted genes including TNF-alpha- and IL-6-induced by lipopolysaccharide (LPS) in mouse macrophages Raw 264.7 cells	[15]

13	*Nypa fruticans* Wurmb.	Arecaceae	MeOH extract of leaf and stem	Antinociceptive	Acetic-acid-induced in mice	[16]

14	*Pandanus foetidus* Roxb.	Pandanaceae	MeOH extract of leaf	Antinociceptive	Acetic-acid-induced in mice	[17]

15	*Pongamia pinnata *(L.) Pierre	Fabaceae	70% EtOH extract of leaf	Antinociceptive and antipyretic activity	Hotplate and tail flick, acetic acid writhing and Randall-Selitto nociceptive tests in mice and brewer's yeast-induced pyrexia in rats	[18]
16	*Pongamia pinnata *(L.) Pierre		70% EtOH extract of leaf	Anti-inflammatory	Carrageenin, histamine, 5-hydroxytryptamine and prostaglandin E-2-induced hind paw edema, kaolin-carrageenan and formaldehyde-induced hind paw oedema, cotton pellet granuloma models of inflammation	[19]
17	*Pongamia pinnata *(L.) Pierre		70% EtOH extract of seed	Antinociceptive, Anti-inflammatory	Carrageenan-induced hind paw oedema and Randall-Selitto nociceptive test in rat	[20]
18	*Pongamia pinnata *(L.) Pierre		PE, CHCl_3_, acetone and EtOH extracts of seed	Antinociceptive, Anti-inflammatory		[21]
19	*Pongamia pinnata *(L.) Pierre		70% EtOH extract of seed	Anti-inflammatory	Bradykinin and PGE-1-induced inflammation, histamine and 5-HT-induced inflammation	[22]

20	*Tamarix indica *Willd.	Tamaricaceae	80% MeOH extract of root	Antinociceptive, Anti-inflammatory	Acetic-acid-induced in mice, using carrageenan induced rat paw oedema	[23]

21	*Derris scandens *(Roxb.) Benth.	Fabaceae	CHCl_3_ extracts of leaf and root and pure compounds	Anti-inflammatory	Carrageenan-induced paw oedema in rats	[24]
22	*Derris scandens *(Roxb.) Benth.		Aqueous extract of stem and pure compounds	Anti-inflammatory	Eicosanoid inhibition	[25]

23	*Ipomoea imperati* (Vahl) Griseb.		EtOH extract of whole plant	Antinociceptive	Acetic-acid-induced and hot plate test in mice	[26]
24	*Ipomoea imperati* (Vahl) Griseb.		MeOH-water extract of leaf	Anti-inflammatory	Mouse ear oedema induced by croton oil, arachidonic acid, cotton pellet-induced granulomas, inhibition of Phospholipase A(2) purified from *Apis mellifera* bee venom	[27]
25	*Ipomoea pes-caprae* (L.) R-Br.	Convolvulaceae	MeOH extract and two fractions of aerial part	Antinociceptive	Acetic-acid-induced and formalin test in mice	[28]
26	*Ipomoea pes-caprae* (L.) R-Br.		Pure compounds	Antinociceptive	Acetic-acid-induced and formalin test in mice	[29]
27	*Ipomoea pes-caprae* (L.) R-Br.		Crude extract and pure compounds	Anti-inflammatory	Inhibition of prostaglandin synthesis *in vitro *	[30]
28	*Ipomoea pes-caprae* (L.) R-Br.		Crude extract	Anti-inflammatory	Carrageenan-induced paw oedema and ear oedema induced in rats by arachidonic acid or ethyl phenylpropiolate, inhibition of prostaglandin synthesis *in vitro *	[31]

29	*Heritiera littoralis *Aiton	Sterculiaceae	Pure compounds	Anti-inflammatory	Nitric oxide (NO) inhibitory effects using RAW 264.7 macrophage cells	[32]

**Table 3:** Analgesic, anti-inflammatory compounds from mangrove plants.

No	Pure compound related to the observed activity	Refs
5	The anti-inflammatory activity of methanolic extract of *Avicennia officinalis *may be due to the presence of the phytoconstituent, betulinic acid	[8]
8	Mimosol D, taepeenin D, taepeenin L, (*E*)-7-hydroxy-3-(4-methoxybenzyl)chroman-4-one, (*E*)-7,8-dihydroxy-3-(4-methoxybenzyl)chroman-4-one, (*E*)-7-hydroxy-8-methoxy-3-(4-methoxybenzyl)chroman-4-one	[11]
10	Calophyllolide	[13]
11	Dehydrocycloguanandin and calophyllin-B	[14]
12	Agallochaol K, agallochaol O, agallochaol P, agallochaol Q, *ent*-17-hydroxykaur-15-en-3-one, *ent*-kaur-15-en-3b,17-diol, *ent*-15,18-dihydroxylabd-8,13*E*-diene	[15]
21	Ovaliflavanone and lupinifolin	[24]
22	3-*γ*,*γ*-dimethylallylweighteone, scandenin and genistein	[25]
26	Glochidone, betulinic acid, *α*-amyrin acetate, *β*-amyrin acetate, isoquercitrin	[29]
27	Eugenol and 4-vinyl-guaiacol	[30]
29	Ergosterol peroxide, 6-*α*-hydroxystigmast-4-en-3-one and stigmast-4-en-3-one	[32]
